# The influence of parents’ history of alcohol use on their university-going children’s drinking habits: A comparative study of students’ drinking habits

**DOI:** 10.4102/sajpsychiatry.v31i0.2508

**Published:** 2025-10-03

**Authors:** Rose Mthembu, Bongani V. Mtshweni

**Affiliations:** 1 Department of Psychology, Faculty of Human Sciences, North-West University, Mahikeng, South Africa; 2 Department of Psychology, College of Human Sciences, University of South Africa, Pretoria, South Africa

**Keywords:** Alcohol abuse, alcohol dependence symptoms, children, harmful alcohol use, hazardous alcohol use, parents, students, university

## Abstract

**Background:**

Parents with a history of alcohol consumption contribute to their children’s drinking behaviour.

**Aim:**

This study aimed to investigate the influence of parents’ history of alcohol use on their children’s drinking habits.

**Setting:**

The study was conducted at a contact learning university in South Africa.

**Methods:**

A cross-sectional quantitative study with 350 undergraduate university students was conducted.

**Results:**

The independent samples *t*-test results revealed that students who had parents with a history of alcohol use (*M* = 4.20, standard deviation [s.d.] = 2.95) scored significantly higher on hazardous alcohol use compared to their counterparts whose parents did not have a history of alcohol use (*M* = 2.47, s.d. = 2.70). The results also showed that students who had parents with a history of alcohol use (*M* = 5.78, s.d. = 4.48) scored significantly higher on harmful alcohol use compared to students with parents who did not have a history of alcohol use (*M* = 2.98, s.d. = 3.66). Moreover, students who had parents with a history of alcohol use (*M* = 4.42, s.d. = 3.20) scored significantly higher on alcohol dependence symptoms than those whose parents did not have a history of alcohol use (*M* = 1.95, s.d. = 2.61).

**Conclusion:**

Parents with a history of alcohol use influence their children’s drinking habits. The observed drinking habits can affect children’s health and interrupt their university studies.

**Contribution:**

Investing in university alcohol prevention programmes could reduce the surge of alcohol abuse among students and promote healthy drinking habits.

## Introduction

Alcohol abuse has pervasive health implications. An estimated 2.6 million deaths recorded globally in 2019 were attributed to alcohol consumption.^1^ Shield et al. noticed that alcohol consumption increases the risk of developing chronic diseases and other health conditions, including neuropsychiatric conditions, cancer, stroke and cardiovascular and digestive diseases.^2^ In addition, alcohol abuse can contribute to mental health conditions such as memory loss, depression and anxiety.^3^ Moreover, alcohol consumption is related to risky sexual behaviours, vulnerability to contracting human immunodeficiency virus (HIV) and other sexually transmitted infections (STIs).^4,5^ Similarly, the consumption of alcohol has been linked to respiratory infections such as tuberculosis and pneumonia, which partly contributed to ‘360 000 deaths and 13 million disability-adjusted life years lost (DALYs) in 2016’.^6(p.1)^

Alcohol abuse has also been associated with social ills. Moss^7^ cautioned that the consumption of alcohol is linked to the risk of injuries and accidents. In 2019, approximately 4.5 million people died from injury-related deaths globally, of which 7% were attributed to alcohol consumption.^8^ Thus, suggesting that consuming alcohol increases the risk of injuries and loss of life. Similarly, research has also revealed that alcohol abuse is linked to crime and violent behaviour, including intimate partner violence and family life disruptions.^9,10^ Alcohol abuse can lead to poor decision-making and put a strain on occupational, social and family relationships, which may lead to long-term psychological distress.

Studies have shown that a maladaptive use of alcohol is prominent among university students in Kenya and Uganda.^11,12^ Similarly, research has indicated that alcohol use among higher education students in South Africa is a growing concern.^13,14^ Kyei et al. revealed that while 65% of South African university students used alcohol, 49% of them abused it.^15^ In addition, scholars found that while 80.6% of a sample of university students in South Africa noticed alcohol was their most used substance,^16^ Maphisa et al. revealed that the risk of substance (alcohol) use disorder of male and female students was 76.1% and 67%, respectively.^17^

South Africa is considered to have one of the highest alcohol consumption rates globally,^18^ thus suggesting that the alcohol consumption rate observed among university students mirrors a societal prevalence. Choi et al. confirmed that the impact of parental drinking on children has contributed to the preservation of intergenerational alcohol use in South Africa.^19^ Brown-Rice et al.^20^ also attested that university students with alcoholic parents have a greater risk of developing alcohol-related problems, depression and becoming dropouts. Moreover, a study found that children of parents with alcohol addiction demonstrated higher rates of alcoholism in comparison to children of parents who do not suffer from alcohol addiction.^7^ Hence, Hansson et al.^21^ proposed an intervention programme for university students who had parents with alcohol problems.

Research conducted in South African universities has revealed that students consumed alcohol in varying patterns, including hazardous and harmful alcohol use.^22,23^ Research has also revealed that students showed dependence symptoms.^14^ Conceptually, hazardous alcohol use refers to a pattern of using alcohol that increases the risk of adverse or harmful consequences for the user.^24^ Friesen et al. noted that this is ‘associated with a clinically meaningful increase in the risk of alcohol-related harm’.^25^ Harmful alcohol use is defined as a drinking pattern that is already causing harm to the individual. This can manifest in various ways, such as liver damage, mental health issues and other ailments.^24^ Alcohol dependence symptoms refer to craving drinks that are concentrated with or contain alcohol and being unable to control one’s drinking.^26^ This is evidenced by not being able to function without alcohol, continual drinking and a preoccupation with the need for alcohol.

By considering the risk factor of parental drinking on their children’s drinking behaviour, this study sought to investigate the influence of parents’ history of alcohol use on hazardous alcohol use, harmful alcohol use and alcohol dependence symptoms among students. The study also aimed to establish whether there were significant differences in students’ hazardous alcohol use, harmful alcohol use and dependence symptoms based on their parents’ history of alcohol use. It is envisaged that the study could assist in highlighting the risk of parental alcohol use on their children’s alcohol use habits and university studies. In addition, it is envisaged that the study could help in proposing suitable intervention programmes for students who abuse alcohol because of exposure to their parents’ alcohol use.

## Literature review on parental history of alcohol use on children’s drinking habits

Literature has linked parents’ alcohol use with their children’s alcohol use. Sondhi et al. argued that while some parents develop ways of passing subtle messages about drinking by introducing samples of alcohol to their children, other children only learn by observation.^27^ Indeed, children tend to imitate and model behaviour observed from their immediate environments. This is supported by social learning theories that posit that children learn about alcohol and how it is used by observing their parents drink.^28^ Jiang^29^ noticed that ‘the greater the amount of time spent with alcohol-using parents, the more likely the children are to use alcohol’, thus implying that parents’ alcohol use is a risk factor for their children’s alcohol use.

South Africa is characterised by widespread substance use among those of a parenting age,^30^ with alcohol being one of the most consumed substances. Rabotata^31^ revealed that young people in a rural village in South Africa who were exposed to adults who drank were likely to engage in drinking behaviours. Ngepah et al.^32^ highlighted the significant role environmental and social factors (i.e., family members who consume alcohol) play in shaping children’s alcohol consumption. Carels et al.^33^ confirmed that modelling the behaviour of family members such as parents, siblings and significant others influenced the use of alcohol among young people, thus suggesting that family exposure to alcohol predisposes children to engage in drinking behaviours.

Alcohol consumption at university has become a ritual that students consider to be part of the experience of higher education.^34^ This experience is associated with a youthful culture characterised by intoxication. Gambles et al.^35^ revealed that university students use alcohol as a mechanism to fit in, secure new friendships and overcome anxieties, which, in turn, reinforces their drinking behaviour. The implication is that drinking alcohol can be used to fulfil a social and psychological role to enable students to cope in a new learning environment. In addition, students may consume alcohol for entertainment or pleasure. Scholars asserted that alcohol is commonly used by students during extramural activities outside of the learning context.^36^ As these periods tend to determine substance-related problems later in life, the need to curb alcohol use among students to inhibit the development of alcohol dependence issues and related health problems is imperative.

Students consume alcohol in varying quantities. Inaç et al.^22^ found that a significant number of students were hazardous drinkers. In addition, Davoren et al.^37^ concluded that in comparison to the general population, the prevalence of hazardous alcohol use was high among university students. Furthermore, scholars have revealed that hazardous alcohol use was high among university students, which was exacerbated when they were surrounded by family members and friends who drank alcohol.^38^ Students who engage in hazardous drinking are negatively affected by their drinking behaviour.^39^ Similarly, harmful alcohol use has been identified to be common among students. Tembo et al.^40^ found that university students consumed harmful levels of alcohol, which was associated with poor mental health outcomes. Research has also revealed that students’ harmful alcohol use poses significant public health issues and exerts an enormous toll on their lives.^34^ Harmful alcohol use among students has been evident through engagement in dangerous activities for fun, injuries, regrettable sexual encounters and memory loss.^41^

Alcohol ‘is a widely used psychoactive substance with dependence properties’.^42^ Knight et al.^43^ revealed that most college students had alcohol dependence symptoms and were, therefore, more likely to receive a dependence diagnosis. Knight et al.^43^ also found that some students sought treatment for their dependence symptoms. Hoeppner et al.^44^ assessed students’ drinking patterns and found that most reported daily drinking, thus suggesting a possible reliance and dependence on alcohol. While alcohol dependence is a challenging issue given the high rates of alcohol consumption among university students, arguably, not all students are dependent on alcohol. However, the risk of abusing alcohol is ever-present given university students’ exposure to alcohol.

University students’ heavy alcohol use leads to a variety of alcohol-related problems, such as psychological distress, suicide, poor academic performance characterised by missing classes, inability to complete assignments and high dropout rates.^45^ As parents’ use of alcohol is suspected to have a potential influence on their university-going children’s drinking behaviour, the study aimed to investigate the influence of parents’ history of alcohol use on hazardous alcohol use, harmful alcohol use and alcohol dependence symptoms among students. In addition, the study sought to examine whether there are differences in students’ levels of hazardous alcohol use, harmful alcohol use and alcohol dependence symptoms based on their parents’ history of alcohol use. Because literature demonstrates that parents’ alcohol consumption behaviour influences their children’s drinking patterns, it was predicted that students’ levels of drinking patterns (i.e., hazardous alcohol use, harmful alcohol use and alcohol dependence symptoms) would vary, as influenced by their parents’ history of alcohol use.

## Research methods and design

This study was conducted with undergraduate students from a contact learning university in South Africa. The university was classified as traditional at the time of the study and most of the students were black females. The convenient sample included 350 students. Of the sample, *n* = 164 (46.86%) were male and *n* = 186 (53.13%) were female. The ages of participants ranged from 18 years to 32 years (*M* = 23.22 years, standard deviation [s.d.] = 3.03 years).

### Data collection

The participants were approached conveniently at their campus residences and asked to complete a paper-based survey. Before completing the survey, R.M. explained the purpose of the study and the contents of the research consent form to the participants. The participants were also allowed to ask questions about the study and the consent form. After they had established what participating in the study would entail, the participants signed the consent form and proceeded to complete the survey.

### Measures

#### Alcohol consumption and/or use

The Alcohol Use Disorder Identification Test (AUDIT) developed by Babor et al.^46^ was employed to assess alcohol use habits among university students. The AUDIT is a 10-item alcohol harm screening measure and can be used in health care, social and educational settings. The AUDIT also provides a framework for intervention to assist drinkers in ceasing or reducing alcohol consumption to avoid harmful consequences. The measure comprises three sub-scales that assess hazardous alcohol use, harmful alcohol use and dependence symptoms. The AUDIT comprises sample items such as ‘How often do you have a drink containing alcohol?’ The items are assessed with a 5-point Likert scale that ranges from 0 (*Never*) to 4 (*4 times or more a week*). The overall score of the AUDIT ranges from 0 to 7 (low risk), 8 to 15 (increasing risk), 16 to 19 (higher risk) and 20 or more (possible dependence). In this study, the reliability coefficients of the AUDIT were as follows: Hazardous alcohol use (α = 0.93), harmful alcohol use (α = 0.80) and dependence symptoms (α = 0.90). The overall measure was found to have a reliability coefficient of α = 0.93.

#### Parents’ history of alcohol use

Parents’ history of alcohol use was measured using a nominal variable. Two categories were used and responded to as follows: Yes = parents with a history of alcohol use; No = parents without a history of alcohol use. Dummy coding was used to assign numerical values to the categories. The ‘Yes’ response was assigned a value of 1, whereas ‘No’ was assigned a value of 0.

### Data analysis

Statistical Package for the Social Sciences Version 29 was employed to analyse the data. The data analyses in the study included descriptive statistics, linear regression and independent samples *t*-tests. The descriptive statistics comprised sample characteristics and correlation analysis, which focused on the association between hazardous drinking, harmful drinking and dependence symptoms. The linear regression model was used to assess the predictive effect of parents’ history of alcohol use on hazardous and harmful alcohol use and dependence symptoms. The independent samples *t*-test analysis entailed assessing differences in students’ hazardous and harmful alcohol use and dependence symptoms based on their parents’ history of alcohol use (i.e., comparisons between parents with a history of alcohol use and parents without a history of alcohol use).

### Ethical considerations

Ethics approval for the study was obtained from the North-West University’s Human Sciences Research Ethics Committee (Ref: NWU-00336-17-A9). Before taking part in the study, participants were made aware that their participation in the study was voluntary and assured of their right to withdraw their participation before data analysis. In addition, they were assured of anonymity and that the information collected would be treated as confidential. The participants who participated in the study signed a consent form before completing the questionnaire.

## Results

The descriptive statistics and bivariate correlations of hazardous alcohol use, harmful alcohol use and dependence symptoms are presented in Table 1. Firstly, the results revealed that hazardous alcohol use significantly and positively correlated with harmful alcohol use (*r* = 0.78, *p* < 0.01). Secondly, the results demonstrated that hazardous alcohol use significantly and positively correlated with dependence symptoms (*r* = 0.79, *p* < 0.01). Finally, the results showed that harmful alcohol use significantly and positively correlated with dependence symptoms (*r* = 0.79, *p* < 0.01).

**TABLE 1:** Descriptive statistics with regard to study variables and bivariate correlations.

Variable	*M*	s.d.	1	2	3
Hazardous alcohol use	3.30	2.95	-	-	-
Harmful alcohol use	4.32	4.28	0.78*	-	-
Dependence symptoms	2.65	2.99	0.79*	0.79*	-

s.d., standard deviation; *M*, mean.

* , *p* < 0.01.

### Linear regression

Linear regression analyses were conducted to determine whether parents’ history of alcohol use could significantly predict hazardous alcohol use, harmful alcohol use and alcohol dependence symptoms. Firstly, hazardous alcohol use was regressed onto parents’ history of alcohol use. Gender and age were controlled to rule out alternative explanations. The model was found to be statistically significant, *F*(1348) = 32.927, *p* < 0.001, and explained 8.6% of the variance in hazardous alcohol use. In addition, parents’ history of alcohol use (*B* = 1.73, *t* = 5.73, *p* < 0.001) significantly predicted hazardous alcohol use among students (Table 2; Model 1). Secondly, harmful alcohol use was regressed onto parents’ history of alcohol use while gender and age were controlled. The results revealed a statistically significant model, *F*(1347) = 41.373, *p* < 0.001, which explained 10.7% of the variance in harmful alcohol use. Additionally, the results revealed that parents’ history of alcohol use (*B* = 2.79, *t* = 6.43, *p* < 0.001) significantly predicted harmful alcohol use among students (Table 2; Model 2). Lastly, alcohol dependence symptoms were regressed onto parents’ history of alcohol use while gender and age were controlled. The results revealed a statistically significant model, *F*(1348) = 22.156, *p* < 0.001, which explained 6% of the variance in alcohol dependence symptoms. Furthermore, the results revealed that parents’ history of alcohol use (*B* = 1.46, *t* = 4.70, *p* < 0.001) significantly predicted alcohol dependence symptoms (Table 2; Model 3).

**TABLE 2:** Effects of parents’ history of alcohol use on hazardous alcohol use, harmful alcohol use and alcohol dependence symptoms.

Outcome variables	Predictive variables	*B*	*SE*	*β*	*t*	*p*	95%CI	
							Lower	Upper
Model 1 Hazardous drinking	Parents’ history of alcohol use	1.73	0.30	0.29	5.73	0.001	1.14	2.33
	Gender	−0.57	0.30	−0.09	−1.86	0.063	−1.17	0.03
	Age	0.09	0.05	0.10	1.98	0.048	0.01	0.19
Model 2 Harmful drinking	Parents’ history of alcohol use	2.79	0.43	0.32	6.43	0.001	1.94	3.65
	Gender	−0.96	0.44	−0.11	−2.19	0.029	−0.83	−0.09
	Age	0.05	0.07	0.03	0.72	0.571	−0.09	0.19
Model 3 Alcohol dependence symptoms	Parents’ history of alcohol use	1.46	0.31	0.24	4.70	0.001	−0.85	2.07
	Gender	−0.48	0.31	−0.08	−1.52	0.127	−1.11	0.13
	Age	0.01	0.05	0.01	0.28	0.778	−0.08	0.11

CI, confidence interval; SE, standard error.

### Independent samples *t*-test

**FIGURE 1:**
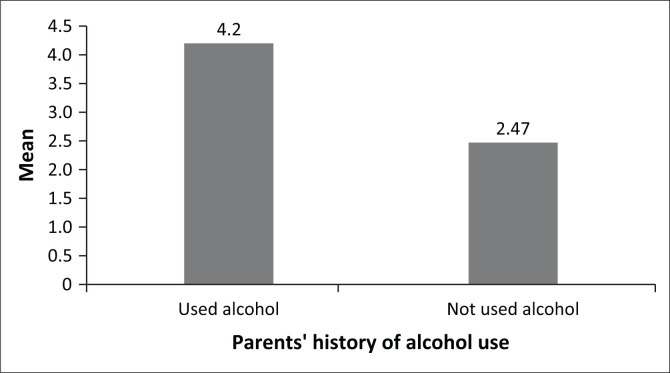
Hazardous alcohol use mean differences.

The first hypothesis tested whether there are variations in students’ levels of hazardous alcohol use based on their parents’ history of alcohol use. Therefore, the levels of hazardous alcohol use between students whose parents had a history of alcohol use and those who did not have a history of alcohol use were compared. The independent samples *t*-test results showed that students who had parents with a history of alcohol use (*n* = 168, *M* = 4.20, s.d. = 2.95) scored slightly higher on hazardous alcohol use than students with parents who did not have a history of alcohol use (*n* = 182, *M* = 2.47, s.d. = 2.70). The observed differences were statistically significant, *t* (338) = 5.71, *p* < 0.01. The results, therefore, suggest that students who have parents with a history of alcohol use are likely to have greater levels of hazardous alcohol use than those with parents without a history of alcohol use. The graphical representation of mean scores is shown in Figure 1.

**FIGURE 2:**
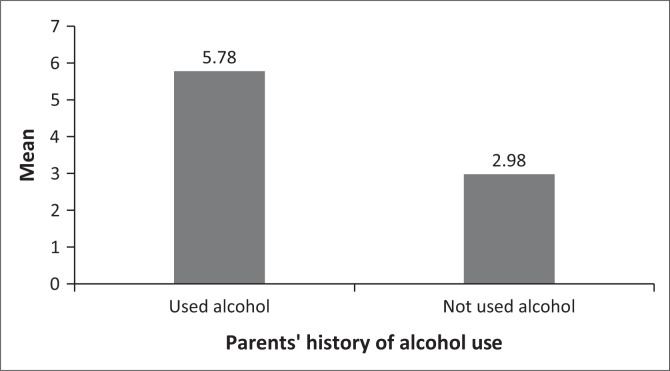
Harmful alcohol use mean differences.

The second hypothesis tested whether there are variations in students’ levels of harmful alcohol use based on their parents’ history of alcohol use. Hence, the levels of harmful alcohol use between students whose parents have a history of alcohol use and those who do not have a history of alcohol use were measured. The independent samples *t*-test results revealed that students who had parents with a history of alcohol use (*n* = 167, *M* = 5.78, s.d. = 4.48) scored slightly higher on harmful alcohol use than students with parents who did not have a history of alcohol use (*n* = 182, *M* = 2.98, s.d. = 3.66). The observed differences were statistically significant, *t* (323) = 6.38, *p* < 0.01. The results, therefore, imply that students who have parents with a history of alcohol use tend to have higher levels of harmful alcohol use than those who have parents without a history of alcohol use. The graphical representation of mean scores is depicted in Figure 2.

**FIGURE 3:**
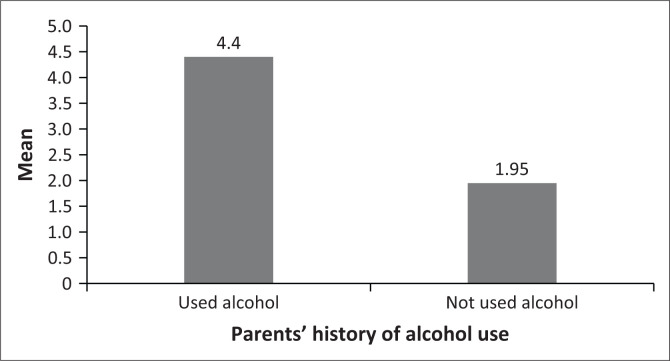
Alcohol dependence symptoms mean differences.

The third hypothesis tested whether there are differences in students’ levels of alcohol dependence symptoms based on their parents’ history of alcohol use. Accordingly, the levels of alcohol dependence symptoms between students whose parents had a history of alcohol use and those with parents who did not have a history of alcohol use were measured. The independent samples *t*-test results showed that students who had parents with a history of alcohol use (*n* = 168, *M* = 4.42, s.d. = 3.20) scored slightly higher on alcohol dependence symptoms than those with parents without a history of alcohol use (*n* = 182, *M* = 1.95, s.d. = 2.61). The observed differences were statistically significant, *t* (323) = 4.67, *p* < 0.01. These results suggest that students who have parents with a history of alcohol use tend to have higher levels of alcohol dependence symptoms than those who have parents without a history of alcohol use. The graphical representation of mean scores is displayed in Figure 3.

## Discussion

Alcohol use has serious psychosocial implications for the user and significant others close to the user. Children are vulnerable and susceptible to parental influence.^38,47^ Similarly, the drinking behaviours of parents can be passed down to children and adversely influence their drinking patterns as they transition to adulthood. Therefore, suggesting that parents’ history of alcohol use can play a crucial role in children’s drinking behaviour. The results of the study revealed that parents’ history of alcohol use significantly predicted hazardous alcohol use, harmful alcohol use and alcohol dependence symptoms. Therefore, corroborating findings from a previous study, which found higher alcohol misuse patterns among children with parents who used alcohol compared to children with parents who do not use alcohol.^48^ Thus, highlighting the risk of exposure to parental alcohol use in engendering unhealthy drinking habits among their children.

This study revealed that students whose parents had a history of alcohol use demonstrated a higher hazardous alcohol use than those whose parents did not have a history of alcohol use, in accordance with previous studies.^38,48^ This implies that parents who drink alcohol have an influence and impose a risk on their children’s drinking behaviour. In addition, the results suggest that when children are exposed to environments and people of influence who drink alcohol, such as parents, their likelihood of becoming hazardous drinkers as they transition to adulthood during university years increases. Ay et al.^38^ attested that the high levels of hazardous alcohol use observed among university students are a result of the usage of alcohol in the family. Alcohol is among the most consumed substances in South Africa, and children are being exposed to a culture of drinking during most of their childhood.^36,49^ Therefore, this study highlights the pervasive repercussions of alcohol use by parents and its deleterious influence on children and their alcohol-use-related behaviours that can manifest during university. Students’ hazardous alcohol use may also have negative educational outcomes^45^ and affect their health. Accordingly, the need to invest efforts to reduce alcoholism in institutions of higher learning as a strategy to increase positive health outcomes and study success is of paramount importance.

Students whose parents had a history of alcohol use were found to have harmful alcohol use patterns in comparison to those whose parents did not have a history of alcohol use. These results are similar to those of previous studies, such as LaBrie et al.,^50^ who demonstrated that a family characterised by a history of alcohol abuse was associated with harmful drinking among students. The results indicate that being raised by parents or in a family of adults who drink alcohol could increase the likelihood of children engaging in harmful drinking, which may manifest during university. Hence, LaBrie et al.^50^ found that students with a family history of alcohol abuse were vulnerable to high levels of alcohol consumption. Similarly, studies conducted among undergraduate students in South Africa demonstrated that they were likely to engage in harmful drinking behaviours.^51,52^ Thus, it is crucial to understand the contributory role of familial factors, specifically parental or family history of alcohol use, towards harmful alcohol use among university students in order to devise appropriate support interventions.

The results of the study revealed differences in alcohol dependence symptoms of students whose parents had a history of alcohol use, scoring significantly higher than those whose parents did not have a history of alcohol use. These findings are similar to those of Knight et al.,^43^ who revealed that students met the diagnoses of alcohol dependence symptoms and that ‘those with parents who drank were more likely to be diagnosed with alcohol dependence’ (p. 267). The implication thereof is that students’ alcohol dependence symptoms can partly be explained by their family’s history of alcohol use, especially when they have been raised by parents who use alcohol. Mbuqa et al.^14^ found that undergraduate students in South Africa tested positive for alcohol dependence symptoms. Alcohol dependence symptoms can signal serious health issues for university students and affect their mental and physical health. Therefore, efforts to educate students about alcohol dependence and addiction are pivotal for addressing alcohol consumption problems.

### Implications for practice

Alcohol abuse and unsafe drinking behaviours are common among university students.^53^ In addition, alcohol poses serious health implications and may disrupt students’ academic outcomes. Scholars have revealed that alcohol abuse among students could lead to physical injury, stress, unprotected sex (i.e., potential risk for contracting STIs), poor academic performance and becoming a dropout.^45^ Accordingly, this study draws attention to the importance of providing support to students by offering intervention strategies to lower the danger of unhealthy drinking behaviours and alcohol abuse in order to increase positive health and academic outcomes.

To address the problem of alcohol abuse in higher learning, it is important that university health centres have dedicated alcohol abuse prevention and educational programmes to increase awareness about alcohol abuse. These educational programmes could focus on promoting moderate and healthy drinking.^51^ In addition, the centres should have dedicated mental health practitioners to help students struggling with alcohol-related mental health problems. Furthermore, these health centres could work collaboratively with internal and external stakeholders, including local health non-governmental organisations, hospitals, student organisations and campus radio stations (e.g., jointly facilitating on-campus outreach day health initiatives) to create awareness.

It is also imperative for universities to regulate drinking behaviour on campus. This could be achieved by establishing a code of conduct or guidelines to minimise unsafe alcohol consumption. Similarly, universities could develop alcohol and substance consumption policies. Rhodes University in South Africa implemented an alcohol policy to promote responsible drinking among students.^54^ Such efforts may reinforce university rules, increase awareness and education, and limit the availability of alcohol use on campus and harm.^54^

It is of paramount importance to promote safe drinking through student orientations. This could be facilitated by members of the university health centres when students attend university induction programmes. In addition, safe drinking tutorials could form part of the first-year university curriculum, during which students could be taught about substance abuse and associated risks. This could help curb unsafe alcohol consumption among students, promote positive health and yield study success.

### Limitations and recommendations

The study has a few limitations. Firstly, a cross-sectional survey design was employed. It is recommended that future studies replicate the study but utilise a longitudinal design to understand students’ drinking behaviours over a longer period so as to devise more suitable alcohol abuse intervention measures. Secondly, self-report measures, which are often susceptible to self-report bias, were used to collect data from the participants. It is recommended that future research employ different data sources to minimise the self-report bias. Thirdly, convenience sampling, which is a non-probability sampling method, was used to recruit participants in this study. Convenience sampling has limited representativeness and generalisability. It is recommended that future research replicate the study using probability sampling methods to increase representativeness and the generalisability of the results. Finally, this study was carried out at a single university in South Africa. It is recommended that future research replicate the study but conduct it at several universities to increase its generalisability.

## Conclusion

The results of the study highlight the influence of parents’ history of alcohol abuse on their children’s drinking behaviour. In particular, the study indicates that the risk of engaging in hazardous, harmful alcohol use and developing dependence symptoms among university students is greater when they have been exposed to alcohol use by parents. The study, therefore, contributes to the literature on parenting practices and alcohol consumption. In addition, the study contributes to the literature on responsible alcohol consumption among students, student health and study success. Although it is acknowledged that institutions of higher learning cannot control students’ pre-university or domestic life, they have a critical responsibility to prevent substance abuse among students and educate them about safe alcohol consumption practices.
